# The NIST Automated Computer Time Service

**DOI:** 10.6028/jres.094.029

**Published:** 1989

**Authors:** J. Levine, M. Weiss, D. D. Davis, D. W. Allan, D. B. Sullivan

**Affiliations:** National Institute of Standards and Technology, Boulder, CO 80303

**Keywords:** automation, computers, delay, digital systems, frequency, propagation delay, telephone, synchronization, time

## Abstract

The NIST Automated Computer Time Service (ACTS) is a telephone time service designed to provide computers with telephone access to time generated by the National Institute of Standards and Technology at accuracies approaching 1 ms. Features of the service include automated estimation by the transmitter of the telephone-line delay, advanced alert for changes to and from daylight saving time, and advanced notice of insertion of leap seconds. The ASCII-character time code operates with most standard modems and computer systems. The system can be used to set computer clocks and simple hardware can also be developed to set non-computer clock systems.

## 1. Introduction

The principal limitation to the accuracy of most methods of time dissemination is the uncertainty in the velocity of propagation of the information through the medium separating the transmitter and receiver. The delay, which is typically on the order of milliseconds, depends both on the physical length of the path and on the group velocity of the signal, and neither of these is well known in general. Either the path length or the group velocity is likely to change with time, so that real-time measurements of the transit time are generally required if the highest accuracy is to be realized. The telephone system provides a unique environment in this respect, since such measurements can be made using simple hardware. Using the telephone system for time dissemination is also desirable since telephone service is already provided to most homes, businesses, and laboratories.

If a passive timing receiver is replaced by an active transponder, the time delay along the path can be determined from the transmission end by sending a pulse to the user and measuring the delay until the echoed pulse returns. Half of this round-trip delay is the time for the signal to reach the user assuming that the communication medium is reciprocal (i.e., that the delay is the same in both directions). This is not always the case for atmospheric paths because of fluctuating multi-path effects and asymmetries in the antennas, and it was not always the case in the past for telephone circuits. It was not uncommon to see telephone connections involving, for example, one-way connection by land-line and the other by satellite. In recent years the telephone carriers have moved away from this practice and now prefer to route both directions of transmission along the same path. As will be seen later in this paper, tests indicate that the telephone path is highly reciprocal.

With this as background and with growing interest in millisecond-level synchronization of computers, NIST has developed a simple telephone system for automated setting of clocks in digital systems. The system makes no demands on the receiver and will function with both passive receivers and active transponders. A passive receiver consists of a modem and a terminal, computer or other display device. An active transponder consists of the same hardware with the additional capability of being able to echo the received messages back to NIST using either hardware or software methods. For either type of system, the modem must conform to either the Bell 103A standard for frequency-shift keying at 300 bits/s or the Bell v212a standard for phase-shift keying at 1200 bits/s. The terminal, computer or display device must recognize the standard ASCII code transmitted with 7 data bits, space parity and 1 stop bit.

The current telephone number for the service is (303) 494-4774. This is not a toll-free number. The following sections describe the service in more detail including discussion of (1) the operation of the service, (2) the reliability of the transmission system and, (3) software and hardware which have been developed for the user end.

## 2. Operation of ACTS

### 2.1 Transmission formats

**Figure 1:**
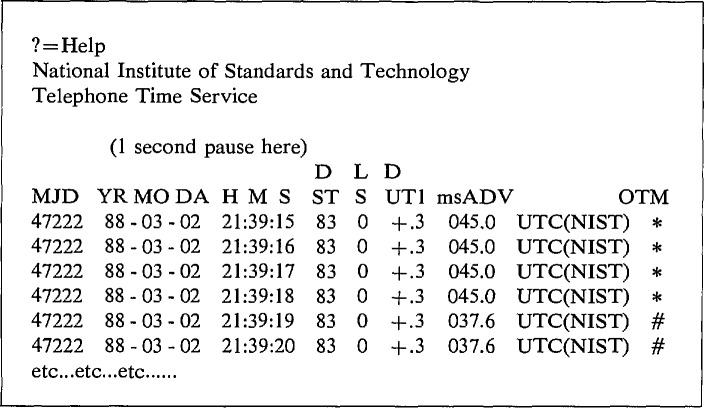
Time code and time marker transmitted by ACTS at 1200 bits/s. The abbreviations are as follows: MJD=Mean Julian Date, YR = Year, MO = Month, DA = Day, H = Hour, M = Minute, S = Second, DST=Daylight Saving Time (a flag meaning that a change is coming), LS = Leap Second (a flag meaning a leap second is to be added), DUT1 = UT1 –UTC (earth rotation time minus coordinated universal time), msADV = milliseconds of advance of the time marker, OTM = On-Time Marker.

The transmission format at 1200 bits/s is shown in figure 1.

- The column labeled MJD is the Modified Julian Day number, which advances by 1 at 0000 Coordinated Universal Time (UTC) every day. The MJD corresponding to 1 January 1989 was 47527.
- The next six numbers give the Coordinated Universal time as: years since 1900, month, day, hour, minute and second. Coordinated Universal Time is the official international time and was formerly called Greenwich mean time.
- The column labeled DST is a flag used to specify if a correction for daylight saving time is required now or is imminent. This flag is valid for most of the continental United States. If DST is 00 then standard time is in effect. If DST is 50 then daylight saving time is in effect. If DST is between 99 and 51 then a transition *to* daylight saving time is approaching. Daylight saving time will be in effect at 2:00 (2:00 a.m.) local time on the day when the count reaches 51. If DST is between 49 and 01 a transition *from* daylight saving time back to standard time is approaching. It will arrive at 2:00 (2:00 a.m.) local time on the day when the counter is 01. In either transition situation, the counter is decremented at 00:00:00 (midnight) UTC every day.
- LS is the leap-second flag and is normally 0. It will be set to 1 to indicate that a leap second is to be added following 23:59:59 UTC on the last day of the current month. This second will be named 23:59:60 UTC, and the second following it will be 00:00:00 of the following day. The LS flag will be set to 2 to indicate that a second is to be dropped at end of the last day of the current month. The second following 23:59:58 UTC will be 00:00:00 UTC of the next day. Added leap seconds are generally required about every 18 months to maintain the coordination of UTC; it is unlikely that seconds will be dropped in the forseeable future.
- DUT1 is the approximate difference between a time scale defined in terms of the rotation rate of the earth (UT1) and UTC. That is, DUT1 = UT1—UTC. The difference is given to the nearest 0.1 s.
- msADV is the advance of the on-time marker in milliseconds. The center of the stop-bit of the on-time marker leaves the transmitter early by this amount so as to arrive at the user on-time. The method used to estimate this parameter and the uncertainty of this estimate are discussed below.
- OTM is the on-time marker. It is either “*” or “#” as discussed below. The center of the stop-bit of this character is intended to arrive at the user at the time specified by the previous characters on the same line.

A help message is available if the user sends a question mark early in the transmission. This, however, preempts the transmission of time information for the rest of that call.

The transmission format at 300 bits/s is similar, but there is not enough time to send the entire message. The transmission consists of the UTC time in hours, minutes and seconds, the current advance in milliseconds and the on-time marker. In the future, alternating halves of the full message may be sent every second together with an on-time marker, so that the full date can be decoded every 2 s.

### 2.2 Modes of Operation of the Service

Depending on the user equipment, the ACTS service provides three modes for checking and/or setting computer clocks.

- In the simplest form of the service, a passive user receives the time code and the on-time marker/character but does not echo the received message. In this case, an advance of 45 ms is used for all transmissions. The OTM should arrive at the user within 100 ms of the correct time unless the connection is routed through a satellite.
- If the user echoes all characters back to NIST, the round-trip line delay of the on-time marker will be used to adjust the advance of subsequent transmissions. The accuracy in this mode should be better than 10 ms and the repeatability is about 1 ms.
- If 300 bit/s modems are used and the user echoes all characters back to NIST, the slower transmission speed means that the full time code is not transmitted, but the measured delay is likely to be more accurate. Our experience indicates that the accuracy will be approximately 1 ms.

In any of these modes, the maximum connection time is 55 s. If all of the lines are busy at any time, the oldest call will be terminated if it has been on line more than 15 s, otherwise the call that first reaches 15 s is disconnected.

### 2.3 Reliability

**Figure 2:**
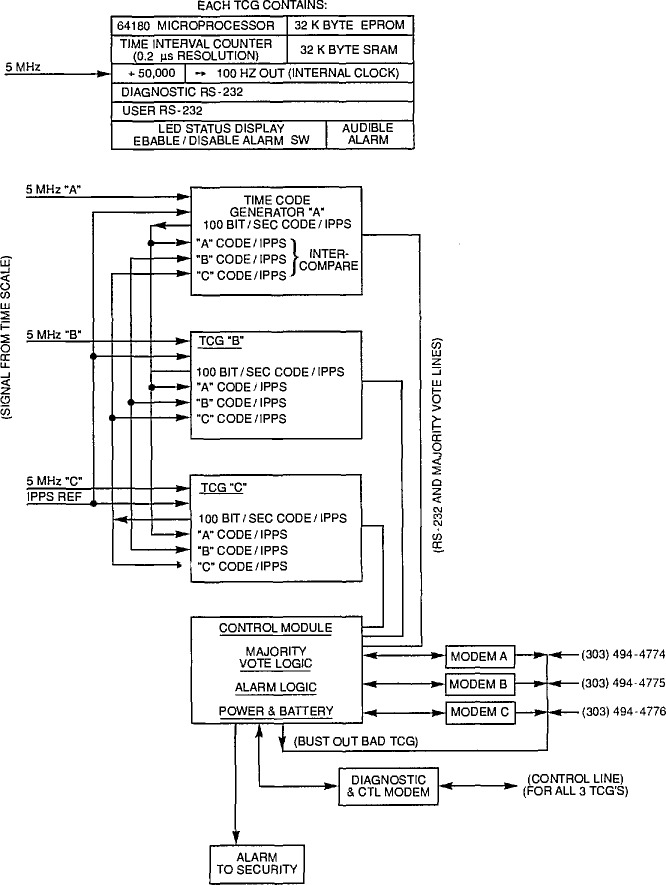
Block diagram of the ACTS transmitter including time-code generators and control module.

To help ensure that ACTS never sends the wrong time, the system has triple redundancy and special self checking to enhance reliability and to increase the volume of calls that can be handled. The basic unit (see fig. 2) consists of three time-code generators, each with a complete system for generating the time-code and disseminating it through a separate modem to one of the telephone lines. Each of the time-code generators receives an independent time signal from a different clock in the NIST time scale. The time code needs to be initialized by an operator, but is automatically updated by the hardware after that. The power supply for the system is backed up by batteries so that the internal clocks do not lose time during a power failure.

Each time-code generator constantly compares its own time code with the codes of the other two, and participates in majority voting on the correctness of these codes. The time code is transmitted at 100 bits/s from each generator to the other two. This code contains all of the information transmitted through the telephone line. Each generator compares the bits of the two incoming codes with its own code and flags a generator as bad if any one disagrees with the other two. The flag of one generator declaring another bad is passed to a central control module as a vote against the offending generator. If any two of the generators vote against any single generator, the loser is taken off line. If all three disagree with one another, all three are disabled.

Each time-code generator receives a timing signal from the other two and compares these signals with its own internal time. If either time difference is larger than 15 *µ*s, this is reported to the control module. Again, the votes are counted by the control module. A vote of two to one takes the loser off line and disagreement among all three disables all of them.

If an individual time-code generator finds either a code or a time error it sounds an audible alarm. If there is total disagreement among the three generators, all three generators are taken off line and the lines to these generators are set so that a caller will receive a busy signal. The system is also connected to a special alarm at the Boulder NIST security office and signals that office if problems develop. This office then contacts one of the system operators, either at work or at home. To facilitate operations and service, the system can be operated remotely using extensive control and diagnostic functions.

The ACTS system can be expanded in three-line units, each operating independently in the manner described above. If an entire three-generator unit is taken off-line, then the effect is to reduce the number of available lines by three. The desired effect is that the system transmits nothing rather than transmitting a code or time marker which is in error.

## 3. User Software and Hardware

### 3.1 Software

We have developed some example ACTS software which runs on a number of popular computers—for IBM PC/XT/AT and compatible systems^1^, for the DEC PDP-11, and for Sun systems. There has been no attempt to be comprehensive in coverage of different computers, but rather to focus on a few example packages which can then be adapted to other machines. The NIST software provides for automated dialing, selection of time zone, selection of mode of operation, echoing of the OTM, setting of the computer clock, archiving of clock offset, and transmission to the port of the computer of a signal which can be used to produce an external time pulse coincident with the OTM. The program is written in a modular fashion so that additional features, such as a graphical presentation of the time-difference data or a more comprehensive statistical analysis of the performance of the local clock, can be added easily. This example software (which includes source code) is available on a $5\frac{1}{4}\text{-in}$, 360-kbyte MSDOS diskette along with instructions for $35.00. To order this software contact:

NIST Office of Standard Reference Materials
B311 Chemistry Building
Gaithersburg, MD 20899
(301) 975-6776

Specify that you want RM 8101, software for Automated Computer Time Service.

### 3.2 Hardware

**Figure 3:**
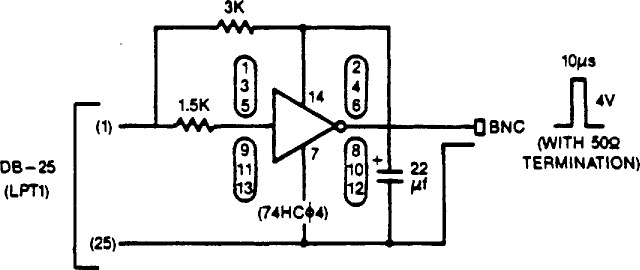
Simple circuit used to obtain a positive pulse coincident with the OTM from any PC/XT/AT compatible computer using the NIST software. The HCMOS invertor can be built directly into a DB-25 male connector.

**Figure 4:**
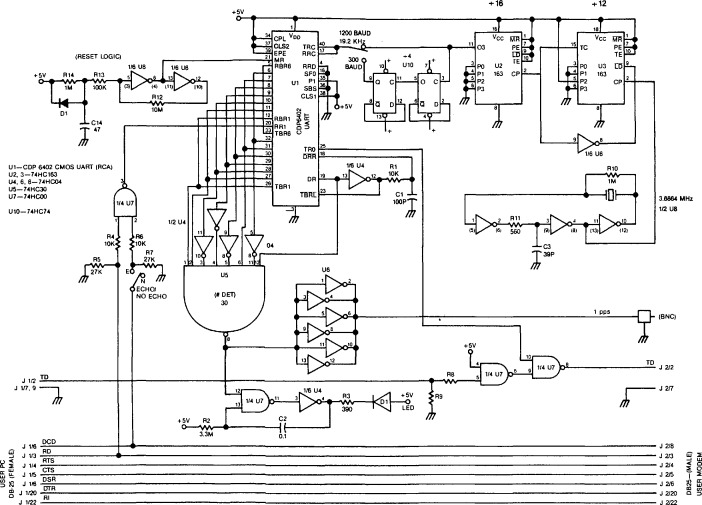
This circuit can be connected directly to a modem or can be bridged across the PC-to-modem cable. An output pulse is generated when the “#” OTM is received. This does not require any special software for the PC.

Using software developed by NIST, the PC/XT/AT compatibles will deliver a signal coincident with the OTM to the parallel printer port. The very simple circuit shown in figure 3 can be used to convert this to a positive pulse. This might be useful for synchronizing an external system or for starting or stopping a counter in a precise measurement of frequency (see sec. 5). A second circuit which we have tested (see fig. 4) echoes all characters from the user and provides an external pulse when the OTM is received. This circuit, which allows ACTS to calibrate the phone line and advance the OTM accordingly, requires an external modem, but does not require a computer. To use this circuit, the user must first manually establish the telephone connection with NIST.

## 4. Timing Accuracy

Since there are many different ways of using the ACTS system, it is not possible to estimate the timing accuracy in general. The following discussion estimates the major contributions to the error budget for many common configurations. Only the most important sources of error are discussed; uncertainties (such as cesium clock performance or variability in the cable delays) whose contribution to the error budget would be less than 1% of the total have been ignored.

### 4.1 Transmitting and Receiving Hardware

The on-time marker is a standard ASCII character which is sent to the user in a standard bit-serial format. Before the start of the transmission, the output line is held at a voltage corresponding to a binary 1. The transmission begins with a binary 0 start bit, the 8 bits that specify the character and a binary 1 stop bit. The 8 character bits are sent with the least significant bit first; the most significant bit, which is sometimes used as a parity bit, is always 0. The binary representations of the transmissions for the two types of on-time marker are 001010100 1 for * and 0 11000100 1 for #. In both cases, the center of the final 1 bit is intended to arrive on time. Each bit is 3.3 ms long at 300 bits/s and 833 μs at 1200 bits/s. The timing of the bits is set by a crystal-controlled oscillator operating at 64 times the bit rate, and the center of the on-time marker can be uncertain by ±0.5 cycle of this oscillator. The corresponding timing uncertainties are thus about 52 μs at a transmission speed of 300 bits/s and 13 μs at 1200 bits/s.

The receiving hardware operates in a similar manner, beginning asynchronously when the leading edge of the start bit is sensed and assembling the bits of the character using a local oscillator which usually operates at 16 times the bit rate. The process ends when the center of the stop bit has been reached. The center of the stop-bit of the on-time marker can be uncertain by ±0.5 cycle of the receiver oscillator, which corresponds to an uncertainty of 208 μs at a transmission speed of 300 bits/s and 52 μs at 1200 bits/s. The transmitter and receiver clocks are not related; the mean value of the combined uncertainty is about 215 μs at 300 bits/s and 54 μs at 1200 bits/s.

### 4.2 Modems

#### 4.2.1 Transmission at 300 bits/s

To transmit and receive data at 300 bits/s, the modems use frequency-shift keying. The originating modem sends 1270 Hz for a binary 1 and 1070 Hz for a binary 0. The receiving modem sends 2225 Hz for a binary 1 and 2025 Hz for a binary 0. Since the line operates in full-duplex with both channels active simultaneously, the frequency slew-rate of the modulator must be controlled to prevent interference between the two channels. In both cases, the receiver must distinguish between two frequencies that are 200 Hz apart in a time that must be shorter than (or at most equal to) the bit-duration time of 3.3 ms. Our measurements indicate that typical discrimination times are about 1 ms; these measurements may vary by as much as 20% depending on signal strength and other factors.

The modem standards do not specify the methods to be used for either the modulator or the demodulator, and some variation in group delay is to be expected among different designs. If the modem does not contain a scrambler, the modem delays are on the order of the bit period and the asymmetry in the two directions is unlikely to exceed 1 ms.

#### 4.2.2 Transmission at 1200 bits/s

These higher speed modems use phase-shift keying to transmit the information. Pairs of bits are combined to form *dibits*; each dibit specifies one of four possible phase shifts that is to be applied to the carrier. The 1200 bit/s data rate is transmitted using one-half that number of phase-transitions/s so that the telephone line and phase detector must operate at 600 Baud. Since the phase of the carrier is significant, the bit-clocks in the transmitting and receiving modems must have the same frequency *and phase.* The asynchronous data bits must be synchronized with this clock, unlike the frequency-shift system described above where the synchronization is with respect to a clock with a frequency much higher than the bit rate.

The bandwidth required by these modems is generally broad enough to require some form of equalization to compensate for variations in the group delay of the telephone circuit with frequency. The equalizer coefficients are adjusted for random input data by transmitting a densely spaced line spectrum when the connection is first established and during idle periods thereafter. These calibration signals are generated by means of digital scramblers—shift-register circuits with feedback whose output is a function of both the current input and the previous transmissions.

The designs of the scramblers, the equalizers and the synchronizers have been widely discussed in the literature, and our interest in them is limited to their effects on the group delay of the transmission, and especially on its asymmetry. The basic delay through a scrambler/de-scrambler combination is about 30 ms and the average value of the offset produced by the synchronization of the asynchronous input data to the transmitter clock is 0.5 clock periods or 417 μs. Although the design of the scrambler is defined by the modem standards, the design of the equalizer is not, and combinations of modems from different suppliers may exhibit significantly different group delays as a result. (This difference is not important in most applications. Some equalizer designs may be better able to cope with poor quality lines, but any two modems using the same scrambler algorithm should be able to communicate with each other in principle.) Since only the sum of the two group-delays is determined by the measurement hardware, the arrival of the on-time marker will be in error by one-half of the difference between the delays in the two directions.

The uncertainties resulting from the 1200 bit/s modems will therefore be composed of three components: one-half of the difference between the scrambler/equalizer delay in the NIST modem and the user’s modem, a synchronizer delay at each end with a mean value of 417 μs and a smaller, slowly fluctuating delay resulting from changes in the automatic equalization.

### 4.3 Delays and Offsets in the Receiver

In order for the NIST hardware to measure the round-trip group delay, the receiver must echo the received message (and particularly the on-time marker). If this is done using a circuit similar to figure 4, for example, the delay between the receipt of the on-time marker and its return to NIST is likely to be stable with a value much smaller than any of the delays discussed above. The delay between the receipt of the on-time marker and the leading edge of the output pulse will also be very small.

If the on-time marker is detected and echoed using software, however, the arrival time of the character is less well defined and may involve both hardware and software delays. The cycle speed of most digital circuits is fast enough so that the sum of these delays is unlikely to be significant in a dedicated single-program environment running with all other interrupts disabled, but is likely to be ill defined in more complex situations.

The arrival of the on-time marker is an asynchronous event with respect to the local clock, and this adds additional complications. We can illustrate these difficulties using a popular type of personal computer. The clock in this type of computer consists of an oscillator with a frequency of 1193180/65536 Hz (about 18.2 ticks/s), and the time is kept as the number of ticks past midnight. It is not possible to read or set the clock to a fraction of a tick. This has two significant consequences:

1. Since there are not an integral number of ticks/s, not all times can exist. For example, 00:00:01.00 is not an integral number of ticks after midnight and therefore can not be set and will never be read. The nearest times are 00:00:00.98 and 00:00:01.04. The average offset between any given time and the nearest time that can be set is 0.5 ticks or about 28 ms.
2. Time intervals are also uncertain by up to 1 tick since the tick fraction when the time is read cannot be known to the program. Again, the average offset is 0.5 ticks (28 ms). This problem increases the uncertainty in measurements of short time intervals and also makes it difficult to estimate the rate of the local clock with respect to NIST using second differences of the local time and UTC(NIST). A typical value for the rate offset of a clock in a small computer is about 1 s/d. Consecutive calibrations of this clock must be separated by at least 10 h if this rate is to be estimated with an uncertainty of no more than 10% using second differences between the local time and UTC(NIST).

If the cycle-speed of the hardware is known, it is possible to interpolate between ticks using short loops to measure the time between the arrival of the on-time marker and the next tick of the local clock in units of the cycle speed of the processor, but this strategy is only useful in a single-user environment when other interrupts are disabled. This capability is incorporated into the latest version of the NIST software for personal computers.

### 4.4 Delays and Offsets in the Transmitter

There are two critical timing operations that are performed by the transmitting hardware: the measurement of the round-trip transit time of the on-time marker, which is used to determine the advance for subsequent transmissions, and the advance itself.

The round-trip transit time is measured by a counter that is started when the on-time marker is sent to the output port for transmission and is stopped when the software recognizes that the appropriate character has just been received. The resolution of the counter is 200 ns. The frequency used to drive the counter is derived from a cesium standard and the uncertainty of this measurement is very small compared to the other effects that have already been discussed.

The uncertainty in the advance is also negligible compared to the other effects. The advance is determined modulo 10 ms using a 100 Hz signal derived from a cesium standard and synchronized to be coincident with UTC(NIST); the remainder is determined using a signal derived from a local 6 MHz crystal oscillator that is also used as the clock for the processor chip that controls the system. This frequency need not have high accuracy since it is used only to measure a small time interval.

### 4.5 Transmission Errors

Transmission errors are usually caused by broadband noise on the telephone line, by echoes, or by cross-talk interference from another telephone circuit. The error rate is too small to estimate accurately, but is unlikely to exceed 10^−6^ unless the telephone connection is unusually noisy.

Since the probability of an error within a single transmission is quite small, NIST transmissions do not contain checksums or other provision for error correction. Successive transmissions are highly redundant, however, and error detection can be included in the design of the receiver without much difficulty. There are several different possibilities to consider, depending on where the error occurs.

1. The on-time marker (* or #) is garbled or lost. If an output pulse was to be generated on receipt of the on-time marker, then no pulse will be sent, and the interval between two consecutive on-time markers will be too long by 1 s. If the on-time marker was to trigger some process (such as setting the local clock), then that process will not be initiated. The loss of the on-time marker can be detected, since its approximate arrival time is known from previous transmissions. A character received at approximately the correct time could be taken as the on-time marker, but neither the hardware nor the software designed by NIST operates in this way.
2. A transmission error converts another character on the line to the on-time marker (* or #). An output pulse will be generated at the wrong time, and this spurious character will be echoed back to NIST too soon so that the measurement of the delay by NIST will be affected. This type of error could be detected by parsing the complete transmitted line and rejecting an on-time marker if it does not occur in the proper context, but neither the hardware nor the software designed by NIST perform this check.
3. An error occurs in one of the digits of the time. If the error transforms the digit into another character that is not legal in context, it is easily detected since the parsing algorithm will fail. If the character in error is still legal in context, the error cannot be detected by examining the line alone. Since the time is composed of about 12 digits and there are 10 legal digits out of 128 possible codes, the probability that an error will transform a digit of the time into another character that is legal in context is less than 10^−6^.

The probability that an error of this kind will result in the time being set incorrectly has been minimized in the NIST software by insisting that the times of two consecutive transmissions differ by exactly 1 s. A general version of such a scheme is quite complex since it must cope with all of the peculiarities of the calendar, and only a simplified version has been implemented. The seconds value from the first line of the pair is decoded and examined. If it is greater than 57, then the comparison is postponed, since the following second may be the first second of a new minute. (Second number 58 is the last second of the current minute if a negative leap-second is imminent, second number 59 is usually the last second of the current minute and second number 60 will be the last second if a positive leap-second is imminent.) If the current second is less than 57, the seconds digits are converted to blanks and the remainder of the date and time are stored in a buffer as received. When the next line is received, the seconds value is extracted and tested for equality with the previous value +1. If this comparison succeeds, the remaining digits of the date and time (with the seconds field converted to blanks in both cases) are compared character by character (all characters must be the same). If either comparison fails a transmission error is assumed, and the process is begun again with the next two lines since the program has no way of knowing which of the two lines is in error. This process continues until two consecutive lines are decoded and are found to be consecutive or until the NIST hardware hangs up the telephone.

This method will detect almost all of the errors that could not be detected by any of the previous methods; it reduces the probability that the time will be set incorrectly to less than 10^−12^.

### 4.6 Results of System Tests

We have evaluated the performance of the ACTS system using both satellite and ground-based telephone circuits. The satellite test was performed between the NIST radio station WWVH in Hawaii and our laboratory in Boulder, Colorado; the ground-based test used a local telephone call in Boulder.

In both tests, we dial the NIST ACTS telephone number, establish a telephone connection, echo all characters back to the transmitter and wait 6 s for the time-delay measurement to stabilize. We use either the circuit of figure 4 or a personal computer running our software to produce a pulse each time the on-time marker arrives at the receiver. We measure the time differences between these pulses and the ticks of a local time standard. These tests are repeated at different times of the day and using different brands of modems, all of which conform to the appropriate standard.

Consecutive measurements over both the satellite and local telephone connections showed a repeatability of ±1 ms at both 300 and 1200 bits/s. This specification is most important for time-interval measurements or frequency calibrations. It is consistent with the value to be expected from the previous discussion, and suggests that both satellite and local telephone connections are reciprocal to a very high degree.

The accuracy of the arrival time of the on-time marker was measured using several different brands of modems. All of the tests used local telephone connections. At 300 bits/s, the offset of the on-time marker was not more than ±2 ms for any modem tested. At 1200 bits/s, different brands of modems resulted in offsets of up to ±7 ms. These values are significantly larger than the repeatability using any one modem, and result from the difference in the group delay between the transmit and receive portions of the modem as discussed above.

The accuracy of the satellite connection to Hawaii was measured using a single modem at both speeds. We measured an accuracy of 1 ms using 300 bits/s and 6 ms using the 1200 bit/s protocol. These values were stable from day to day to within the repeatability quoted above, and suggest that the satellite path is also reciprocal to a very high degree.

## 5. Applications

One of the most important applications for ACTS is the maintenance of accurate time within a digital computer or some digital hardware with a microprocessor. This might be required, for example, for tagging of business transactions or scientific data. Where a number of computers independently tag events with the date and time and then share their information, it may be especially important to know that the clocks in all of the computers are set to the same time.

A second application is the measurement of frequency. Here pulses coincident with the OTM are used to start and then stop a counter which counts the output of the oscillator under test. If the start and the stop pulses are separated by one day, then the system can yield a frequency-measurement accuracy of about one part in 10^8^.

## 6. Discussion and Conclusions

NIST is now committed to long-term operation of a new Automated Computer Time Service. The format is now fixed, except for a change which will modify the 300 bit/s format to transmit the entire time/date message every 2 s. The system will be expanded as the number of calls increases. The telephone number for the service is (303) 494-4774. All of the lines will be connected to a telephone rotary switch so that they can be reached by dialing this number. This number is not toll-free and might be changed in the future.

The system was designed to make operation at the user’s end particularly simple. With telephone-line delay measured at the NIST end, the user needs only a modem and a computer or some other digital system to access NIST time at accuracies of up to 1 ms. Example software and hardware has been developed by NIST, and the software is available for a small charge.

The system can be used to set digital clocks, or to perform frequency calibration at an accuracy of one part in 10^8^ for a one day measurement. For systems that are already connected to the telephone line, little effort is needed for setting time. We might imagine development of a number of products which could take advantage of this. For example, a digital clock and microprocessor built into a telephone receiver could provide for a clock which is “always right.” Such a telephone could be designed to distribute time to other clocks within a business, school or factory. Automated calling could be done during night hours when the telephone charges are least expensive. If this approach is used, we ask that some effort be directed toward spreading the calling times out over a broad period so that the ACTS system is not heavily taxed in some narrow time frame (e.g., midnight).

The uncertainties in the method are fully discussed in section 4. Using a transmission speed of 1200 bits/s, the major systematic offset is the asymmetry in the delays of the pair of modems. This offset is largely removed at 300 bits/s. At either speed, the path between the receiver and the transmitter contains several asynchronous to synchronous conversions. The jitter inherent in such processes is the largest single contributor to the error budget; it cannot be removed using the current components.

These uncertainties result from the use of standard modems and protocols which were chosen to make the system widely available and simple to use; more specialized, higher accuracy versions of the protocol, using special-purpose hardware, are under consideration. The ultimate limitation of the method is the lack of perfect reciprocity in the telephone connection. Preliminary measurements suggest that the group delay in a local telephone loop is stable and reciprocal to about 20 μs, suggesting that both the repeatability and the bias might be improved by a factor of 50 using appropriate transmission hardware.

## Footnotes

1 Certain commercial equipment, instruments, and materials are identified in this paper in order to adequately specify the experimental procedure. In no case does such identification imply recommendation or endorsement by the National Institute of Standards and Technology, nor does it imply that the material, instruments, or equipment identified is necessarily the best available for the purpose.

